# Palliative radiotherapy indications during the COVID-19 pandemic and in future complex logistic settings: the NORMALITY model

**DOI:** 10.1007/s11547-021-01414-z

**Published:** 2021-09-27

**Authors:** Francesco Cellini, Rossella Di Franco, Stefania Manfrida, Valentina Borzillo, Ernesto Maranzano, Stefano Pergolizzi, Alessio Giuseppe Morganti, Vincenzo Fusco, Francesco Deodato, Mario Santarelli, Fabio Arcidiacono, Romina Rossi, Sara Reina, Anna Merlotti, Barbara Alicja Jereczek-Fossa, Angelo Tozzi, Giambattista Siepe, Alberto Cacciola, Elvio Russi, Maria Antonietta Gambacorta, Marta Scorsetti, Umberto Ricardi, Renzo Corvò, Vittorio Donato, Paolo Muto, Vincenzo Valentini

**Affiliations:** 1 Dipartimento di Diagnostica per Immagini, Radioterapia Oncologica ed Ematologia, Fondazione Policlinico Universitario “A. Gemelli” IRCCS, UOC di Radioterapia Oncologica, Roma, Italia; 2 Università Cattolica del Sacro Cuore, Istituto di Radiologia, Roma, Italia; 3 Department of Radiation Oncology, Istituto Nazionale Tumori-IRCCS-Fondazione G. Pascale, Napoli, Italy; 4 Radiotherapy Oncology Centre, Santa Maria Hospital, Terni, Italy; 5 Department of Medicine and Surgery, University of Perugia, Perugia, Italy; 6 Dipartimento di Scienze Biomediche, Odontoiatriche e delle Immagini Morfologiche e Funzionali, Università di Messina, Messina, Italy; 7 Radiation Oncology, IRCCS Azienda Ospedaliero - Universitaria di Bologna, Bologna, Italy; 8 DIMES, Alma Mater Studiorum - Bologna University, Bologna, Italy; 9 Radiotherapy Oncology Department, IRCCS CROB, Rionero In Vulture, Italy; 10 Radiotherapy Unit, Gemelli Molise Hospital, Campobasso, Italy; 11 Radiotherapy Unit, Ospedale San Camillo de Lellis, Rieti, Italy; 12 Radiation Oncology, Azienda Ospedaliera Santa Maria di Terni, Terni, Italy; 13 Palliative Care Unit IRCCS Istituto Romagnolo per lo Studio dei Tumori (IRST) Dino Amadori, Meldola, Italy; 14 Struttura Complessa di Radioterapia, Azienda Sanitaria Ospedaliera S. Croce e Carle, Cuneo, Italy; 15 Department of Oncology and Hemato-oncology, University of Milan, Milan, Italy; 16 Division of Radiotherapy, IEO European Institute of Oncology, IRCCS, Milan, Italy; 17 Department of Radiation Oncology, ICS Maugeri SpA SB-IRCCS, Pavia, Italy; 18 Radiotherapy and Radiosurgery dept, IRCCS Humanitas Research Hospital, Rozzano (Milan), Italy; 19 Department of Biomedical Sciences, Humanitas University, Pieve Emanuele (Milan), Italy; 20 Radiation Oncology Unit, Department of Oncology, University of Torino, Torino, Italy; 21 Radioterapia Oncologica, IRCCS Ospedale Policlinico San Martino, Genova, Italy; 22 Dipartimento di Scienze della Salute (DISSAL), Università degli Studi di Genova, Genova, Italy; 23 Dipartimento Oncologia e Medicine Specialistiche, UOC Radioterapia, A.O. S. Camillo ‑ Forlanini, Rome, Italy

**Keywords:** Palliative Radiotherapy, COVID-19, Palliation, QoL, Consensus, Clinical Indication, Clinical Care Model, Guidelines

## Abstract

**Introduction:**

The COVID-19 pandemic has challenged healthcare systems worldwide over the last few months, and it continues to do so. Although some restrictions are being removed, it is not certain when the pandemic is going to be definitively over. Pandemics can be seen as a highly complex logistic scenario. From this perspective, some of the indications provided for palliative radiotherapy (PRT) during the COVID-19 pandemic could be maintained in the future in settings that limit the possibility of patients achieving symptom relief by radiotherapy.

This paper has two aims: (1) to provide a summary of the indications for PRT during the COVID-19 pandemic; since some indications can differ slightly, and to avoid any possible contradictions, an expert panel composed of the Italian Association of Radiotherapy and Clinical Oncology (AIRO) and the Palliative Care and Supportive Therapies Working Group (AIRO-palliative) voted by consensus on the summary; (2) to introduce a clinical care model for PRT [endorsed by AIRO and by a spontaneous Italian collaborative network for PRT named “La Rete del Sollievo” (“The Net of Relief”)]. The proposed model, denoted “No cOmpRoMise on quality of life by pALliative radiotherapy” (NORMALITY), is based on an AIRO-palliative consensus-based list of clinical indications for PRT and on practical suggestions regarding the management of patients potentially suitable for PRT but dealing with highly complex logistics scenarios (similar to the ongoing logistics limits due to COVID-19).

**Material and Methods:**

First, a summary of the available literature guidelines for PRT published during the COVID-19 pandemic was prepared. A systematic literature search based on the PRISMA approach was performed to retrieve the available literature reporting guideline indications fully or partially focused on PRT. Tables reporting each addressed clinical presentation and respective literature indications were prepared and distributed into two main groups: palliative emergencies and palliative non-emergencies. These summaries were voted in by consensus by selected members of the AIRO and AIRO-palliative panels. Second, based on the summary for palliative indications during the COVID-19 pandemic, a clinical care model to facilitate recruitment and delivery of PRT to patients in complex logistic scenarios was proposed. The summary tables were critically integrated and shuffled according to clinical presentations and then voted on in a second consensus round. Along with the adapted guideline indications, some methods of performing the first triage of patients and facilitating a teleconsultation preliminary to the first in-person visit were developed.

**Results:**

After the revision of 161 documents, 13 papers were selected for analysis. From the papers, 19 clinical presentation items were collected; in total, 61 question items were extracted and voted on (i.e., for each presentation, more than one indication was provided from the literature). Two tables summarizing the PRT indications during the COVID-19 pandemic available from the literature (PRT COVID-19 summary tables) were developed: palliative emergencies and palliative non-emergencies. The consensus of the vote by the AIRO panel for the PRT COVID-19 summary was reached. The PRT COVID-19 summary tables for palliative emergencies and palliative non-emergencies were adapted for clinical presentations possibly associated with patients in complex clinical scenarios other than the COVID-19 pandemic. The two new indication tables (i.e., “Normality model of PRT indications”) for both palliative emergencies and palliative non-emergencies were voted on in a second consensus round. The consensus rate was reached and strong. Written forms facilitating two levels of teleconsultation (triage and remote visits) were also developed, both in English and in Italian, to evaluate the patients for possible indications for PRT before scheduling clinical visits.

**Conclusion:**

We provide a comprehensive summary of the literature guideline indications for PRT during COVID-19 pandemic. We also propose a clinical care model including clinical indications and written forms facilitating two levels of teleconsultation (triage and remote visits) to evaluate the patients for indications of PRT before scheduling clinical visits. The normality model could facilitate the provision of PRT to patients in future complex logistic scenarios.

## Introduction

The COVID-19 pandemic has challenged healthcare systems worldwide over the last few months, and this is still ongoing [1, 2]. Although some restrictions have been removed depending on the country, it is not certain when the pandemic is going to be over for certain. Radiation oncologists (ROs) will be forced to face the pandemic for an unknown time interval. Two main approaches have been adopted to control COVID-19: suppression and mitigation, the latter being the most frequently adopted [3]. The mitigation approach imposes the need for indications for how and when to delay or omit radiotherapy (RT). Alternatively, hypofractionated RT schedules, which adequately manage different clinical settings, have been proposed to reduce the number of interactions and physical contact in hospitals (for both patients and patients) while delivering effective treatments [4–7].

National and international guidelines and expert opinions about RT indications and prescriptions have been provided for primary malignancies (e.g., head and neck [6] or gastrointestinal [7]). More often, palliative RT (PRT) indications during the COVID-19 pandemic scenario are dealt with using reports focused on primary malignancies. To the best of our knowledge, very few guidelines have been specifically dedicated to PRT, and in some cases, these are limited to particularly relevant palliative presentations (e.g., bone metastases [8]). Although the level of priority of PRT has frequently been the object of discussion [3, 9–11], it remains one of the primary aims of RT. Once the COVID-19 pandemic has concluded, many of the RT indications currently modified due to pandemic issues could not be further considered for most primary tumors. Conversely, in some situations (e.g., patients admitted in hospice; patients living at high distance from an RT department; less-resourced developing countries), the issue of patients suitable for PRT but dealing with complex logistic settings and thus subject to limitations in their possibility to achieve symptom relief by PRT will surely persist. Therefore, some of the indications provided for PRT during the COVID-19 pandemic could be safely and effectively maintained in these peculiar settings (since they are currently clinically accepted).

This paper has two aims: (1) to provide a summary of the indications for PRT during the COVID-19 period. Since some published guidelines are slightly different, in order to harmonize the suggestions, an expert panel composed of the Italian Association of Radiotherapy and Clinical Oncology (AIRO) and the Palliative Care and Supportive Therapies Working Group (AIRO-palliative) voted by consensus on the summary. (2) To introduce a clinical care model for PRT [endorsed by AIRO and by a spontaneous Italian collaborative network for PRT named “La Rete del Sollievo” (“The Net of Relief”)]. The proposed model, denoted “No cOmpRoMise on quality of life by pALliative radIoTherapY" (NORMALITY), is based on an AIRO-palliative consensus-based list of clinical indications for PRT and on practical suggestions regarding the management of patients potentially suitable for PRT but dealing with highly complex logistics scenarios (similar to the ongoing logistics limits due to COVID-19).

## Material and methods

The two aims of this project were handled separately and progressively. The first aim (1) was to summarize the PRT clinical indications during the COVID-19 pandemic from the available RT literature. In particular, we aimed to (1.1) provide a summary (PRT COVID-19 summary) of the indications and guidelines for PRT during the COVID-19 period and to (1.2) have the AIRO expert team group vote by consensus on the PRT COVID-19 summary.

Our second (2) aim was to create the NORMALITY clinical care model (“No cOmpRoMise on quality of life by pALliative radIoTherapY"). In particular, we aimed to (2.1) provide a set of PRT indications for patients dealing with complex logistic scenarios (strongly limiting their possibility of receiving PRT beyond its given clinical indication) in order to (2.2) provide practical advice and supportive materials to optimize the clinical management of these patients by RT departments.

### Summarize the PRT clinical indications from the available COVID-19 RT literature

####  Create the PRT COVID-19 summary

A systematic literature search based on the PRISMA approach was performed by two radiation oncologists (ROs; RDF and VB). The search was performed using PubMed. We applied the following medical subject headings (MeSH) and keywords such as: “Radiotherapy,” “Radiation Therapy,” “Radiation Oncology,” “Palliative,” “Palliative Radiotherapy,” “COVID-19,” and “SARS-COV2.” The detailed Medline search strategy is reported in Appendix.

**Fig. 1:**
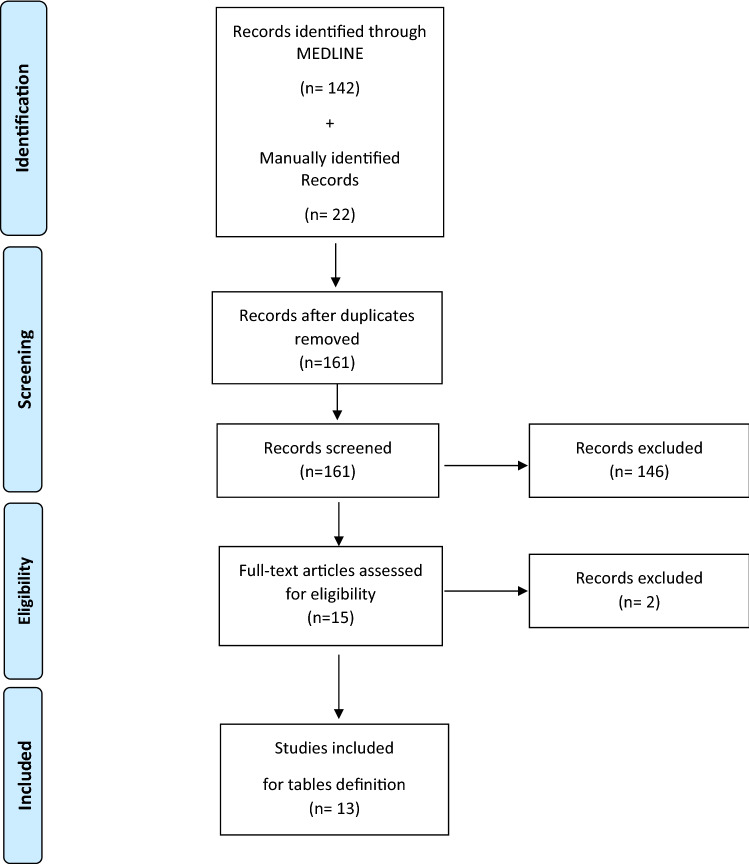
PRISMA Literature Search

For the first literature search, other documents were added by a manual search performed by a third RO (SM). The review was strictly composed of full-text publications that were written in English and reported clinical indications for PRT to be applied during the period of the COVID-19 pandemic. The literature search was conducted on April 26, 2020. All types of publications were initially considered, including surveys, letters, and editorials, provided that the prescriptive indications for PRT were clearly reported. Papers reviewing literature or personal considerations and not directly addressing a prescriptive indication were excluded. Reports of congress abstracts and book chapters were excluded. Reports providing clinical PRT indications and those that did not undergo a peer-review process were also excluded. No specific time restrictions other than those implicitly related to the COVID-19 pandemic period were applied. An independent literature revision was made by a different RO who supervised the summary consolidation process (FC). Eligible citations were retrieved for full-text review. Figure 1 illustrates the PRISMA workflow.

To homogenize the summary output, the clinical presentations discussed in the documents were collected into two main groups: emergencies and palliative non-emergencies. A retrospective collection of each clinical presentation was organized into these two groups within multiple clinical presentation subgroups (defined as “clinical presentation items,” CPI).

Three information categories were extracted from each selected document: (i) the main (preferable) PRT prescriptive indication; (ii) the alternative (secondary) PRT prescriptive indication; (iii) additional statements in the document per each subgroup clinical presentation not strictly indicating a PRT prescription but specifically aimed at the considered subgroup topic.

A document was excluded by a certain clinical presentation subgroup if none of the three categories were addressed, but possibly included for other subgroup topic indications.

A group of four separate ROs double-checked the different sections of the PRT COVID-19 summary to confirm the correspondence of the data extraction (FA, AT, GS, and AC).

#### AIRO expert team consensus vote on the PRT COVID-19 summary

An expert panel of 14 AIRO members, which was not involved in any of the previously described phases, was asked to vote on, in a single round, the consensus of each of the reported PRT indications. Consensus was addressed by four options: 1 = “strongly agree,” 2 = “agree,” 3 = “disagree,” and 4 = “strongly disagree”. Consensus was based on all of the indications from each paper (i.e., main + alternative PRT indication + additional statements), thus preventing experts from only agreeing or disagreeing with specific parts of the summarized papers.

For each paper reported in the summary, the results were analyzed by a single vote and coupled as either a “positive consensus vote” (i.e.: 1 + 2) or “negative consensus vote” (i.e.: 3 + 4).

As for other experiences [5], an agreement or disagreement threshold ≥ 66% was required for each item to reach a consensus and a threshold of ≥ 80% was required for a strong consensus.

### NORMALITY (“No cOmpRoMise on quality of life by pALliative radIoTherapY") clinical care model

The spontaneous network named “*La Rete del Sollievo*” (www.laretedelsollievo.net) (i.e. *Net of Relief,* NOR), which is set up at the Department of Radiation Oncology of Fondazione Policlinico A. Gemelli IRRCS (Rome, Italy), promotes palliative radiation oncology clinical care models and shares research projects in collaboration with the AIRO-palliative panel. Under the endorsement of the AIRO, the NOS aimed to create a clinical care model for patients with an indication for PRT who are dealing with high complexity logistic scenarios that limit their possibility of receiving a regular PRT schedule.

#### Provide PRT indications (through consensus vote by the AIRO expert team)

From the evidence base of the PRT COVID-19 summary, a table of PRT indications for patients in complex logistic settings (potentially other than the COVID-19 pandemic) was set up for the NORMALITY clinical care model. The table “Normality model of PRT indications” followed the structure of the PRT COVID-19 summary. The AIRO expert panel voted in two rounds of consensus using the same previously described methodology. In this case, we also added the opportunity to provide comments and alternative indications for the first voting round only. After the revision of the first-round votes and comments, a final version of the table was voted on once more. Analysis of the consensus was performed as previously described. Only the results of the final consensus round were considered in the analysis.

#### Provide practical advice and supportive materials for the NORMALITY clinical care model

To establish the practicalities of the NORMALITY clinical care model, the definition of the workflow was addressed based on the available literature indications [9, 12], some currently ongoing practices among the Radiation Oncology departments of the AIRO experts involved in the project, and through discussion among the AIRO experts. The core concept was the advantage of a preliminary evaluation of the patient’s indications ahead of a live visit. During the live visit, the PRT prescription would be confirmed, potentially including (within the same day) the RT simulation and the first (or single) session administration. Moreover, two types of forms aiding practical patient management were prepared: one to perform the first general patient data collection and allow for triage in palliative settings, and the second to aid the offline evaluation of patients ahead of clinical visits.

## Results

### PRT COVID-19 summary + AIRO expert team consensus vote

From the search results of 161 documents, 13 papers were selected for data extraction [4–9, 13–19]. Globally, 19 clinical presentation items (CPIs) were identified. For “Emergencies,” the following CPIs were extracted from the literature and considered: metastatic epidural spinal cord compression (MESCC); hemostasis (including hemoptysis); and mediastinal syndrome.

For “Palliative Non-Emergencies,” the following CPIs were extracted from the literature and considered: painful bone metastasis; non-painful bone metastasis; bone oligometastases suitable for (stereotactic body radiation therapy) SBRT; retreatment of painful bone metastasis; adjuvant (post-surgery) bone metastasis radiotherapy; pain symptoms NOT associated with bone metastases; symptomatic hematological malignancies; other oligometastases suitable for SBRT (lung); other oligometastases suitable for SBRT (liver); other oligometastases suitable for SBRT (adrenal); other oligometastases suitable for SBRT (lymph-node asymptomatic); brain metastases (N° 1–4); brain metastases (N° 5–10); brain metastases (N° > 4), poor Karnofsky performance status (KPS), meningeal involvement; primary symptomatic brain tumor, poor KPS; and postoperative brain metastases.

In total, 61 question items to be voted on were extracted from the papers including the 19 CPIs. Table 1 presents the PRT COVID-19 summary for palliative emergencies. Table 2 presents the PRT COVID-19 summary for palliative non-emergencies, along with the consensus results. References cited by selected papers are also reported in the table, if specifically related to trials [5, 20–50]. The average agreement was over the agreement threshold, with only 10/61 question items from the different evidence having an agreement below 60% and five that were below 50%. The latter was related to single evidence regarding painful bone metastases, bone metastases suitable for SBRT, adjuvant treatment of bone metastases, other than pain symptoms related to lung primary, and SBRT for adrenal lesions.

**Table 1:** PRT Covid-19 Summary: palliative emergencies

Emergencies						
Reference		Main Prescriptive Indication	Alternative	Additional Statement (if any)	% Consensus Vote*	
					A = Agreement (1 + 2)	
					D = Disagreement (3 + 4)	
					SA = Strong Agreement (1)	
					SD = Strong Disagreement (4)	
***E1 Metastatic Epidural Spinal Cord Compression (MESCC)***						
QE1a	[9]	8 Gy/1fx8Gy [Maranzano [19]]	–	• Requires multidisciplinary discussion with neurosurgery, and evaluation of factors including degree of spinal cord compression and presence or absence of spinal instability	A = 100% [SA = 100%]	D = 0% [SD = 0%]
				• Similar impact on OS and post-RT motor functions than multifractions		
				• Retreatment is safe		
QE1b	Curigliano [16]	–	–	• RT is urgent	A = 100% [SA = 80%]	D = 0% [SD = 0%]
QE1c	Thureau [8]	8 Gy/1fx8Gy	–	• Surgical treatment should theoretically be preferred if possible and for all pt with a life expectancy of more than few months	A = 70% [SA = 30%]	D = 30% [SD = 0%]
				• Adjuvant RT after surgery for MESCC can be postponed for 4 to 12 weeks		
				• In cases where surgical treatment is contraindicated or not appropriate, RT should be arranged without delay		
				• The simplest conformal RT techniques should be used		
				• MESCC is likely the only instance justifying urgent management of a COVID + patient		
QE1d	Simcock [14]	6-10 Gy/1fx6-10 Gy [ICORG 05–03 [20], TROG 96.05 [21]]	–	• Prefer 3D	A = 80% [SA = 10%]	D = 20% [SD = 0%]
***E2 Hemostasis (including Hemoptysis)***						
QE2a	Tchelebi [7]	• **Esophageal cancer bleeding:** 6–8 Gy/ 1fx 6-8 Gy	–	• **Gastric cancer bleeding:** RT should be strictly reserved for palliation of symptoms in pts with gastric cancer at the present time	A = 80% [SA = 20%]	D = 20% [SD = 0%]
		• **Gastric cancer bleeding:** 6–8 Gy/ 1fx 6-8 Gy (with anti-emetic)				
QE2b	[9]	• **Pelvic malignancies bleeding:** 14.8 Gy/4fx/3.7BID	–	**Pelvic malignancies bleeding pt Covid + :** Avoid BID	A = 80% [SA = 20%]	D = 20% [SD = 0%]
		• **Pelvic malignancies bleeding, pt Covid + :** 20 Gy/5fx4Gy				
QE2c	Wu [13]	**Hemoptysis:** 20 Gy/5fx4Gy	–	Palliative lung radiation should be deferred when possible, otherwise reserved for pt with life-threatening complications such as high-volume hemoptysis	A = 80% [SA = 30%]	D = 20% [SD = 10%]
		• 17 Gy/2fx8.5 Gy^**§**^				
		• 10 Gy/1fx10Gy				
QE2d	Hahn et al. [63]	**Pelvic bleeding:** 8 Gy/1fx8Gy	–	–	A = 80% [SA = 40%]	D = 20% [SD = 0%]
QE2e	Combs [15]	**Bleeding** 8 Gy /1fx8Gy (not further specified)	–	–	A = 60% [SA = 30%]	D = 40% [SD = 0%]
QE2f	Thomson [6]	**H&N** bleeding: o Scenario 1- Early Pandemic—Risk mitigation	–	–	A = 70% [SA = 30%]	D = 30% [SD = 0%]
		• 8 Gy/1fx8Gy				
		• 20 Gy/5fx4Gy				
		• 44.4 Gy/12fx3,7 Gy				
		o Scenario 2- Late Pandemic—Severe shortage of RT capacity				
		• 8 Gy/1fx8Gy				
		• 20 Gy/5fx4Gy				
QE2g	Simcock [14]	**Esophageal bleeding:** • 12 Gy/4fx3Gy BID [SHARON project [23]]	**Esophageal bleeding:** 15 Gy/3fx5Gy [SHARON project]**§**	**Esophageal bleeding:** • Prefer 3D	A = 80% [SA = 30%]	D = 20% [SD = 0%]
		• 18 Gy/3fx6Gy Day (Q) 0, 7, 21 (weekly) (Adapted from other sites) [25]		**Pelvic/GI bleeding:**		
		**Pelvic/GI bleeding:**		• Prefer 3D		
		• 20-24 Gy/5-6fx4Gy		• Prefer 3D		
		• 18 Gy/4fx4.5 Gy BID				
		[SHARON project [23]]				
		• 14.8 Gy/4fx3.7 Gy BID (Repeat q2-4 wks to total 44.4 Gy in 3 courses) [QUAD SHOT- RTOG 8502 [26, 27]]				
		• 18-24 Gy/3fx6-8 Gy Day 0, 7, 21 [25]				
		• 18-24 Gy/3fx6-8 Gy Day 0, 7, 21 [25]				
***E3 Mediastinal Syndrome***						
QE3a	Yerramilli [9]	**SVC syndrome Airway Obstruction:** • 17 Gy/2fx8.5 Gy (each, weekly) [Sundstrom [31]]	–	Multidisciplinary discussion may be recommended	A = 100% [SA = 70%]	D = 0% [SD = 0%]
		• 20 Gy/5fx4Gy				
QE3b	Guckenberger	o **NSCLC-Early Phase** of the COVID-19 pandemic (risk mitigation):	–	Order reported for **“NSCLC Early Phase”** follows the highest consensus reported in the paper	A = 80% [SA = 50%]	D = 30% [SD = 0%]
		2. 8–10 Gy/1fx 8–10 Gy 20 Gy/5fx 4 Gy				
		o **NSCLC -Later phase** of the COVID-19 pandemic: (lack of RT resources and need for patient triage) 8-10 Gy/1fx 8-10 Gy				
QE3c	Wu [13]	**Superior vena cava syndrome:**	–	Palliative lung RT should be deferred when possible, otherwise reserved for patients with lifethreatening complications such as superior vena cava syndrome	A = 70% [SA = 40%]	D = 30% [SD = 0%]
		• 17 Gy/2fx8.5 Gy^**§**^				
		**§(Authors do not specify in text/table but the reference report the schedule as “*weekly*”)** [24] [Rodrigues]				
		• 10 Gy/1fx10Gy				
		**SCV Syndrome/Lung**				
QE3d	Simcock [14]	**Cancer:**	–	Prefer 3D	A = 90% [SA = 30%]	D = 10% [SD = 0%]
		• 8–10 Gy/1fx8-10 Gy				
		• 17 Gy/2fx8.5 Gy (weekly) [33] [MRC]				

^§^(Authors do not specify in text/table but the reference report the schedule as “*weekly*”) [Rodrigues [24]]

^**§**^ Note: the schedule reported in the paper do not corresponds to Sharon Project schedule

^*^Consensus Vote: 1 = Strongly Agree; 2 = Agree; 3 = Disagree; 4 = Strongly Disagree

*MESCC* Metastatic Epidural Spinal Cord Compression; *fx* fraction; *OS* overall Survival; *RT* Radiotherapy; *pt* patient; *BID* bis in die; *Q* schedule repetition interval; *QoL* quality of life; *SBRT* stereotactic body *RT* mets: metastases; *wks* weeks; *PEG* percutaneous endoscopic gastrostomy; *WBRT* whole brain RT; *TMZ* Temozolamide; *mth* months; *IMRT-SIB* Intensity modulated RT—Simultaneous integrated boost

**Table 2:** PRT Covid-19 Summary: palliative non-emergencies

Palliative (Non-Emergencies)						
Reference		Main Prescriptive Indication	Alternative	Additional Statement (if any)	% Consensus Vote*	
					A = Agreement (1 + 2)	
					D = Disagreement (3 + 4)	
					SA = Strong Agreement (1)	
					SD = Strong Disagreement (4)	
***P1 Painful bone metastasis***						
QP1a	Thureau [8]	8 Gy/1fx8Gy	6-10 Gy/1 fx6-10 Gy	• Adapt the medical treatment as much as possible and avoid palliative RT in pt controlled by level 1 to 3 oral analgesics	A = 70% [SA = 40%]	D = 30% [SD = 0%]
				• Palliative RT remains an important option for patients experiencing significant pain, diminished QoL and reduced autonomy by bone metastases, especially if it enables a reduction in the need for daily nursing care		
				• The simplest conformal		
				RT techniques should be used		
				• Other that 8 Gy should be avoided		
QP1b	Simcock [14]	8 Gy/1fx8Gy	–	• Evaluate Omission	A = 40% [SA = 10%]	D = 60% [SD = 20%]
				• If RT is for symptom relief then it is best to ensure that all other options have been fully explored e.g. maximizing analgesia or bisphosphonates in the case of bone pain		
QP1c	Combs [15]	• 8 Gy/1fx8Gy	–	–	A = 60% [SA = 30%]	D = 40% [SD = 0%]
		• 20 Gy/ 5 fx4Gy				
		• 21 Gy/ 3 fx7Gy				
QP1d	Yerramilli [9]	8 Gy/1fx8Gy	–	• If pts have life expectancy of days to weeks: refer to Best supportive Care	A = 50% [SA = 10%]	D = 50% [SD = 20%]
				• If pts have life expectancy of longer than weeks, but not emergency: delay RT		
				• pt with less urgent symptoms (able to wait planning) single-fraction SBRT may be considered		
QP1e	Curigliano [16]	–	–	• Advanced breast cancer (ABC): RT is urgent if pts not responding to pharmaceutical interventions	A = 80% [SA = 20%]	D = 20% [SD = 10%]
***P3 Bone Oligometastases Suitable for SBRT***						
QP3a	Thureau [8]	–	Single fraction (16 to 24 Gy) SBRT for Retreatment of Symptomatic MESCC	• Evidence for using SBRT in oligometastatic is too low to be considered in the current situation	A = 90% [SA = 30%]	D = 10% [SD = 0%]
				• It is often possible to postpone this treatment for a few weeks, especially for hormone sensitive tumors		
QP3b	Simcock [14]	–	–	• Omit RT	A = 20% [SA = 0%]	D = 80% [SD = 80%]
QP3c	Combs [15]	SBRT 1–5 fx (not further specified)	–	–	A = 80% [SA = 10%]	D = 20% [SD = 10%]
***P4 Retreatment of painful bone metastasis***						
QP4a	Thureau [8]	8 Gy/1fx8Gy	–	• Waiting a minimum of 6 weeks after completion of the initial RT	A = 90% [SA = 50%]	D = 10% [SD = 0%]
				• The simplest conformal RT techniques should be used		
***P5 Adjuvant (post-surgery) bone metastasis radiotherapy***						
QP5a	Thureau [8]	30 Gy/10 fx3Gy	20 Gy/4 or 5fx 5 or 4 Gy	• RT may be postponed or performed secondarily in case of progressive post-operative signs	A = 80% [SA = 50%]	D = 20% [SD = 0%]
QP5b	Simcock [14]	–	20 Gy/4or5fx 5or4Gy	• Omit RT	A = 40% [SA = 0%]	D = 60% [SD = 20%]
***P6 Pain NOT associated to Bone Mets**** (e.g.: direct infiltration, primary pancreatic; H&N; Lymph-node infiltrating surrounding structures, etc.)*						
QP6a	Thomson [6]	**H&N**: If restricted RT department resources single fraction could be used: 8 Gy/1fx8Gy	**H&N**: If restricted RT department resources: 20 Gy/5fx4Gy	• Symptomatic benefit and chance of cure are two of the top three factors determining which patients should start RT within 1–3 wks	A = 70% [SA = 30%]	D = 30% [SD = 0%]
				• Do not postpone RT initiation of HNSCC radiotherapy by more than 4–6 wks		
				• If Covid + pt delay RT until clinical recover		
				• Use a more hypofractionated schedule if restricted RT department resources		
QP6b	Combs [15]	**H&N**:	–	–	A = 60% [SA = 20%]	D = 40% [SD = 10%]
		14 Gy/4fx 3.5 Gy BID (repeated Q 4 weeks interval × 2 times) [QUAD SHOT- RTOG 8502 [26, 27]]				
QP6c	Hahn et al. [63]	**H&N + Gyn + Melanoma**:	–	–	A = 80% [SA = 40%]	D = 20% [SD = 0%]
		• 8 Gy/1fx8Gy				
		• 18–24/3fx6-8 Gy Q 0–7-(21 if needed) [25, 29, 36]				
QP6d	Tchelebi [7]	• Pain by **primary Esophageal + HCC**: 6–8/1fx6-8 Gy	–	–	A = 80% [SA = 20%]	D = 20% [SD = 0%]
		• Pain by **primary Pancreas**: 8–10/1fx8-10 Gy				
QP6e	Rathod [18]	**SCLC/NSCLC**:	–	–	A = 90% [SA = 20%]	D = 10% [SD = 0%]
		• 8–10 Gy/1fx8-10 Gy [IAEA [37]]				
		• 16 Gy/2fx 8 Gy (1 week apart) [IAEA [37]]				
***P7 Other than Pain symptoms NOT associated to Bone Mets**** (e.g.: obstruction, etc.)*						
QP7a	Thomson [6]	(**H&N**) If restricted RT department resources single fraction could be used: 8 Gy/1fx8Gy	(**H&N**) If restricted RT department resources: 20 Gy/5fx4Gy	• Do not postpone RT initiation of HNSCC RT by more than 4–6 wks	A = 60% [SA = 30%]	D = 40% [SD = 0%]
				• If Covid + pt: delay RT until clinical recover		
				• Use a more hypofractionated schedule if restricted RT department resources		
QP7b	Combs [15]	**H&N**:	–	–	A = 70% [SA = 20%]	D = 30% [SD = 0%]
		14 Gy/4fx 3.5 Gy BID (repeated Q4 weeks interval × 2 times) [QUAD SHOT- RTOG 8502 [26, 27]]				
QP7c	Simcock [14]	**H&N**:	**H&N**: 18or24Gy/3fx6or8 (1 fx/week) Prefer 3D or IMRT	–	A = 70% [SA = 10%]	D = 30% [SD = 0%]
		• 36 Gy/5fx6Gy				
		(2 fx/week)				
		• 30 Gy/6fx6Gy				
		(2 fx/week) [HYPO trial [38]]				
QP7d	Hahn et al. [63]	**H&N + Gyn + Melanoma**:	–	–	A = 60% [SA = 40%]	D = 40% [SD = 0%]
		• 8 Gy/1fx8Gy 18–24 Gy/3fx6-8 Gy Q 0–7-(21 if needed)				
QP7e	Simcock [14]	**Esophageal dysphagia**:	**Esophageal dysphagia**: 15 Gy/3fx5Gy [SHARON project]**§ § Note: the schedule reported in the paper do not corresponds to Sharon Project schedule**	–	A = 60% [SA = 0%]	D = 40% [SD = 0%]
		• 12 Gy/4fx3GyBID [SHARON project [23]]				
		• 18 Gy/3fx6 (1 fx/week)				
QP7f	Tchelebi [7]	**Esophageal Dysfagia**:20 Gy/5fx4Gy	–	• RT is preferred over either an esophageal stent or percutaneous endoscopic gastrostomy (PEG) tube placement in order to avoid consumption of limited operative supplies and aerosolization of the virus secondary to intubation	A = 90% [SA = 50%]	D = 10% [SD = 0%]
QP7g	Tchelebi [7]	**Pancreas Symptomatic** (non-pain): 8–10 Gy/1fx8-10 Gy	–	–	A = 70% [SA = 20%]	D = 30% [SD = 0%]
QP7h	Rathod [18]	**SCLC/NSCLC**:	–	–	A = 70% [SA = 30%]	D = 30% [SD = 0%]
		• 8-10 Gy/1fx 8–10 Gy				
		• 16 Gy/2 fx 8 Gy (1 week apart) [IAEA [37]]				
QP7i	Guckemberger [5]	**NSCLC** Early Phase (Risk Mitigation):	–	• **NSCLC** Early Phase (Risk Mitigation): do not postpone RT of 4–6 weeks	A = 70% [SA = 20%]	D = 30% [SD = 0%]
		1.17 Gy/2 fx 8.5 Gy ^**§**^		• Postpone or interrupt RT if pts is or became Covid +		
				• Order for “**NSCLC Early Phase**” follows the highest consensus reported in the paper		
		2.8–10 Gy/1fx 8–10 Gy				
		3.20 Gy/5fx 4 Gy				
		**NSCLC** Later Phase (Lack of RT Resources):				
		• 8–10 Gy/1fx 8–10 Gy				
QP7j	Wu [13]	–	–	• Lung tumors: palliative lung radiation should be deferred	A = 20% [SA = 0%]	D = 80% [SD = 20%]
***P8 Symptomatic Haematological Malignancies (non-emergencies)***						
QP8a	Yahalom 4	• **Symptomatic aggressive NHL** (no chemo options) Life expectancy > 3 months 25 Gy/5fx5Gy	–	• Consider omitting RT when the risk of severe outcomes from COVID-19 infection (aged ≥ 60 years and/or presence of serious underlying health conditions) outweigh the benefit of RT; where alternatives can be offered e.g. optimizing pain control	A = 100% [SA = 40%]	D = 0% [SD = 0%]
		• **Symptomatic aggressive NHL** (no chemo options) Life expectancy < 3 months: 8 Gy/1fx8Gy				
		• **Symptomatic multiple myeloma** (No cord compression): 8 Gy/1fx8Gy				
		• **Symptomatic multiple myeloma** (Cord compression): 20 Gy/5fx4Gy				
		• **Symptomatic indolent lymphoma** (No cord compression): 4 Gy/1fx4Gy				
		• **Symptomatic indolent lymphoma** (Cord compression): 20 Gy/5fx4Gy				
		• **Myeloid sarcoma/leukemia** -Cranial leptomeningeal disease: 8 Gy/2fx4Gy				
		• **Myeloid sarcoma/leukemia**—Focal leptomeningeal spine disease, and symptomatic chloroma outside the CNS: 12 Gy/3fx4Gy				
QP8b	Simcock [14]	**Palliative Lymphoma, low grade**: 4 Gy/1 fx4Gy	–	–	A = 90% [SA = 40%]	D = 10% [SD = 0%]
***P9 Other Oligometastases Suitable for SBRT (Lung)***						
QP9a	Combs [15]	–	–	SBRT 1–5 fx (not further specified)	A = 50% [SA = 20%]	D = 50% [SD = 0%]
***P10 Other Oligometastases Suitable for SBRT (Liver)***						
QP10a	Combs [15]	–	–	SBRT 1–5 fx (not further specified)	A = 50% [SA = 20%]	D = 50% [SD = 0%]
QP10b	Tchelebi [7]	**Colorectal Primary**	–	–	A = 60% [SA = 30%]	D = 40% [SD = 0%]
		• **For small, non-central lesions:** 16–30 Gy in 1fx				
		• **For lesions near the biliary tree:** 48–60 in 3–5 fx				
***P11 Other Oligometastases Suitable for SBRT (Adrenal)***						
QP11a	Combs [15]	–	–	SBRT 1–5 fx (not further specified)	A = 40% [SA = 30%]	D = 60% [SD = 0%]
***P12 Other Oligometastases Suitable for SBRT (Lymph-node asymptomatic)***						
QP12a	Combs [15]	–	–	SBRT 1–5 fx (not further specified)	A = 50% [SA = 30%]	D = 50% [SD = 0%]
***P13 Brain metastases (N° 1–4)***						
QP13a	Yerramilli [9]	SRS (not further specified)		• In pt with good performance SRS for all or dominant lesion cause of morbidity	A = 80% [SA = 30%]	D = 20% [SD = 0%]
				• To delay or avoid whole brain		
QP13b	Combs [15]	**1–10 Brain Mets with good performance status:** *“single fraction”*			A = 60% [SA = 10%]	D = 40% [SD = 10%]
		• 18 Gy/1fx18Gy				
		• 20 Gy/1f × 20 Gy				
QP13c	Simcock [14]	**1–3 Brain Mets, good KPS, no extracranial disease** 15–20 Gy/1fx15-20 Gy		• SRS	A = 100% [SA = 60%]	D = 0% [SD = 0%]
***P14 Brain metastases (N° 5–10)***						
QP14a	Simcock [14]	**Palliation WBRT:** 20 Gy/5fx4Gy [RTOG QUARTZ [43]]	–	• 3D WBRT	A = 100% [SA = 30%]	D = 0% [SD = 0%]
				• A routine option in UK, Europe, Asia, Canada, and Australia. Established in RTOG dose escalation studies		
QP14b	Combs [15]	**1–10 Brain Mets with good performance status:** *“single fraction”*		• SRS	A = 50% [SA = 20%]	D = 50% [SD = 10%]
		• 18 Gy/1fx18Gy				
		• 20 Gy/1f × 20 Gy				
***P15 Brain metastases**** (N° > 4), poor KPS, meningeal involvement*						
QP15a	Yerramilli [9]	**Multiple brain metastases or leptomeningeal disease WB:**	–	• For patients with urgent indications, progressive neurologic symptom	A = 90% [SA = 40%]	D = 10% [SD = 0%]
		• 20 Gy/5fx4Gy		• For patients in whom longer term survival is expected, in order limit neurocognitive complications		
		• 30 Gy/10fx3Gy		• In patients with limited prognosis, the QUARTZ study demonstrated similar rates of overall survival and QoL with steroids and best supportive care alone		
QP15b	Combs [15]	**Life expectancy > 3 mth:** WBRT 20 Gy/5fx4 Gy	–	**Poor performance status** Evaluate BSC with critical view of steroids [RTOG QUARTZ^10^]	A = 100% [SA = 50%]	D = 0% [SD = 0%]
QP15c	Curigliano [16]	–	–	**RT is urgent for the following situations:** Treatment of brain and leptomeningeal metastases	A = 80% [SA = 20%]	D = 20% [SD = 10%]
QP15d	Simcock [14]	**Brain metastasis Palliation, poor Prognosis:** 12 Gy/2fx6Gy	–	**CNS mets from NSCLC needing WBRT:**	A = 70% [SA = 10%]	D = 30% [SD = 0%]
				• Best supportive care including steroids		
				• Omit RT		
				[RTOG QUARTZ [43]]		
				**Brain Mets Palliation, poor Prognosis:**		
				• Prefer 3D		
***P16 Primary symptomatic Brain tumor, poor KPS***						
QP16a	Combs [15]	**Glioblastoma KPS < 60:** 25 Gy/5fx5Gy; (No TMZ) [Roa 2004 [44]]	–	• **Glioblastoma KPS < 50; age > 70yy** TMZ mono (MGMT methylated) or BSC [Malmstrom [45]]	A = 90% [SA = 30%]	D = 10% [SD = 0%]
QP16b	Simcock [14]	**GBM, poor KPS: Age ≥ 50, KPS 50–70, or Age ≥ 65 KPS 50–100:** 25 Gy/5fx5Gy No TMZ	–	• **GBM, poor KPS Age ≥ 50, KPS 50–70, or Age ≥ 65 KPS 50–100:** Prefer 3D; CTV 2 cm margin as per EORTC	A = 90% [SA = 30%]	D = 10% [SD = 0%]
				• **Glioblastoma Age > 60, methylated** TMZ only Standard RT associated with poor outcomes		
QP16c	Noticewala [19]	**GMB, very poor PS KPS < 50:**	–	• **GMB, very poor PS KPS < 50:** Alternatively, consider: Best supportive care or TMZ with omission of RT	A = 100% [SA = 10%]	D = 0% [SD = 0%]
		• 34 Gy/10fx3.4 Gy [Malmstrom [45]]		• **Recurrent GBM:** not generally recommend re-irradiation Systemic therapies if considered reasonable. Therapies may include, but are not limited to temozolomide, bevacizumab, lomustine, and others		
		• 25 Gy/5fx5Gy [Roa 2015 [46]]				
***P17 Postoperative Brain Mets***						
QP17a	Combs [15]	**Postoperative SRS of resection cavity:**	–	*The dose depends on target diameter:	A = 90% [SA = 40%]	D = 10% [SD = 0%]
		• 35 Gy/7fx5Gy or		• < 2.0 cm		
		• 20-24 Gy/1fx20-24 Gy* [Brown [47]] or		• 2 ≤ 2.9 cm #The dose depends on target size (in cc):		
		• 16 Gy/1fx16Gy		• ≤ 10 cc		
		• 14 Gy/1fx14Gy		• 10.1–15 cc		
		• 12 Gy/1fx12Gy [Mahajan [48]]#		• > 15 cc		

^§^(Authors do not specify in text/table but the reference report the schedule as “*weekly*”) [MRC [32]]

^*^Consensus Vote: 1 = Strongly Agree; 2 = Agree; 3 = Disagree; 4 = Strongly Disagree

*MESCC* Metastatic Epidural Spinal Cord Compression; *fx* fraction; OS: overall Survival; *RT* Radiotherapy; *pt* patient; *BID* bis in die; *Q* schedule repetition interval; *QoL* quality of life; *SBRT* stereotactic body RT; *mets* metastases; *wks* weeks; *PEG* percutaneous endoscopic gastrostomy; *WBRT* whole brain RT; *TMZ* Temozolamide; *mth* months; *IMRT-SIB* Intensity modulated RT—Simultaneous integrated boost

### NORMALITY (“No cOmpRoMise on quality of life by pALliative radIoTherapY") clinical care model

**Fig. 2:**
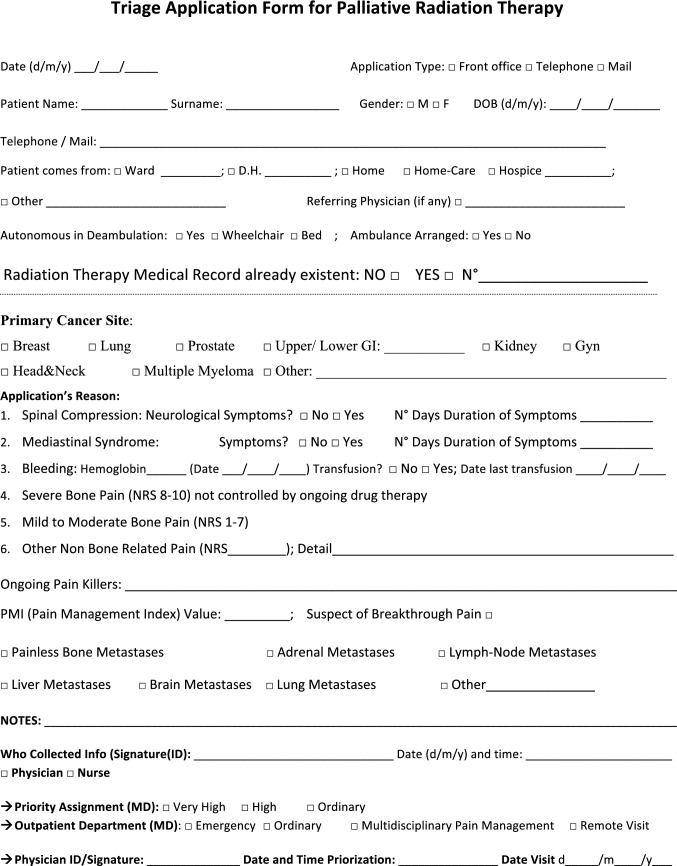
Triage application form for Palliative Radiation Therapy (English Version)

**Fig. 3:**
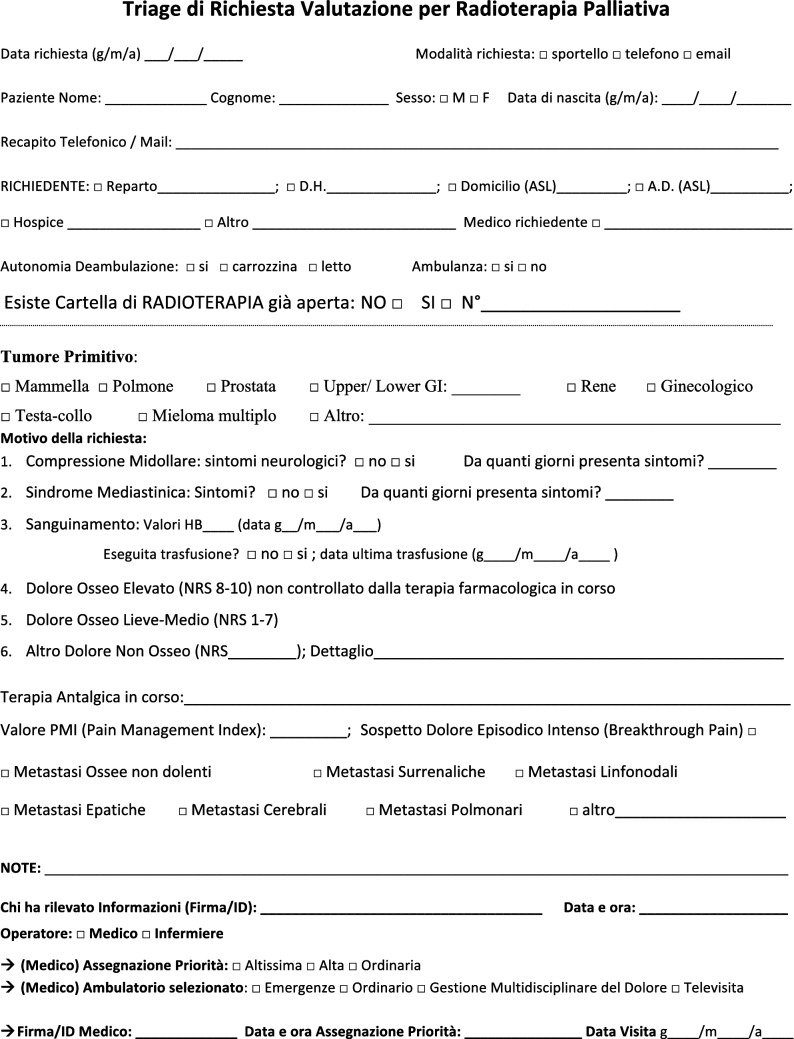
Triage application form for Palliative Radiation Therapy (Italian Version)

**Fig. 4:**
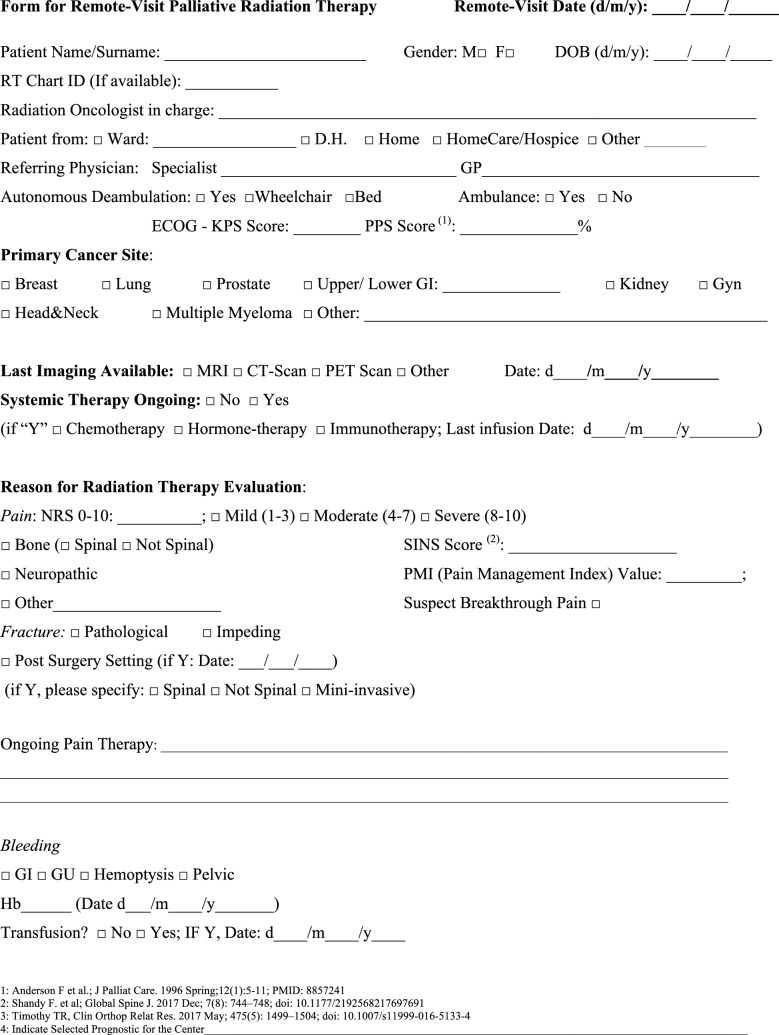

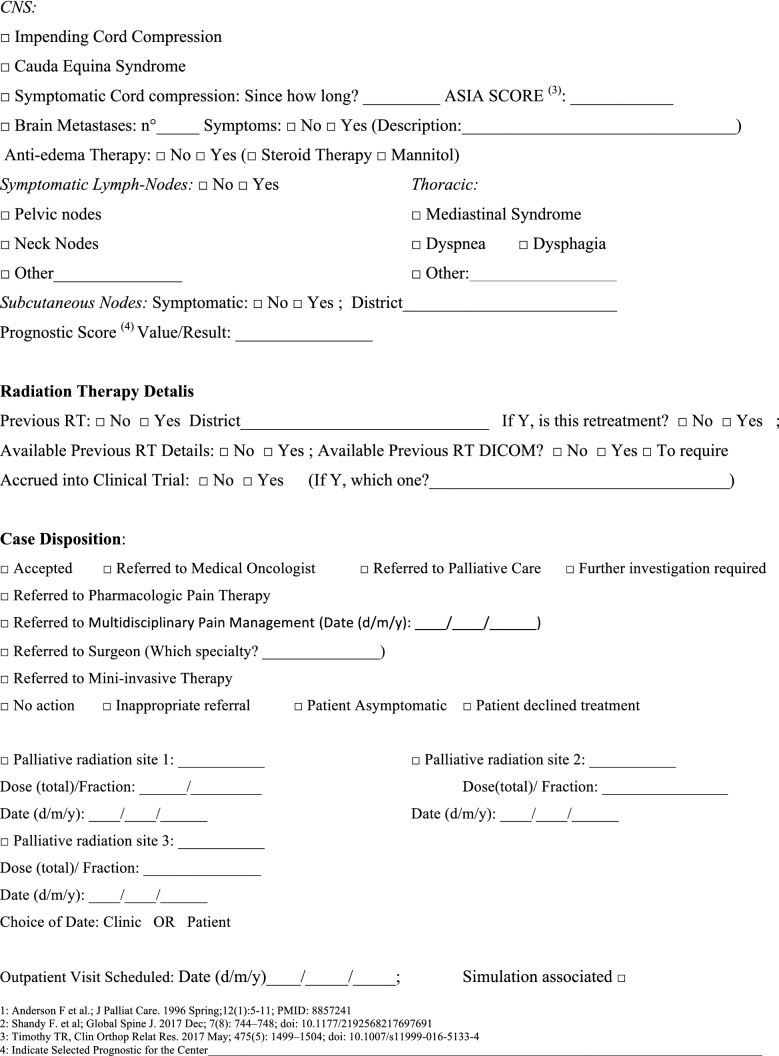
Form for remote-visit for Palliative Radiation Therapy (English Version)

**Fig. 5:**
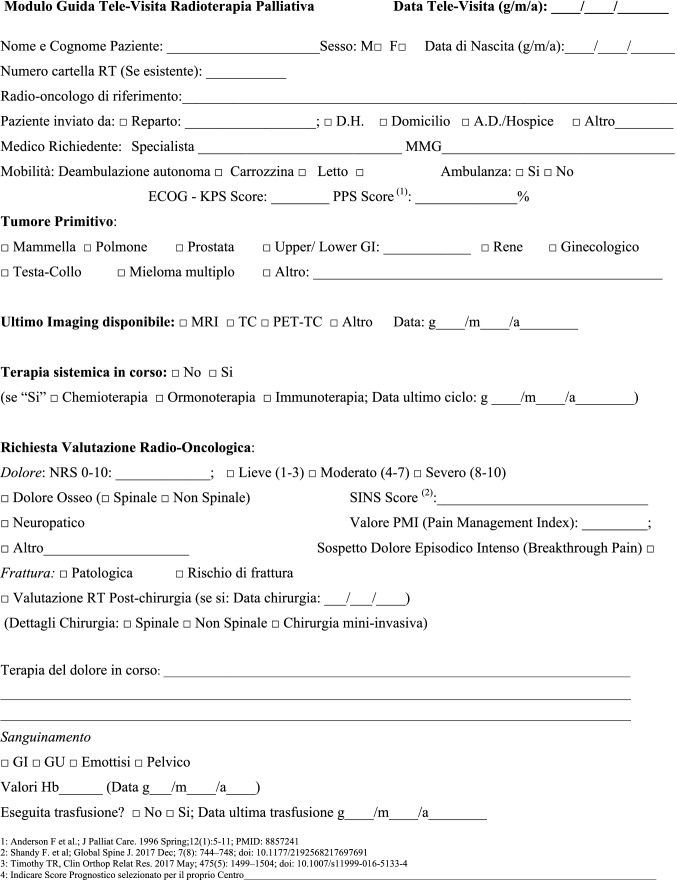

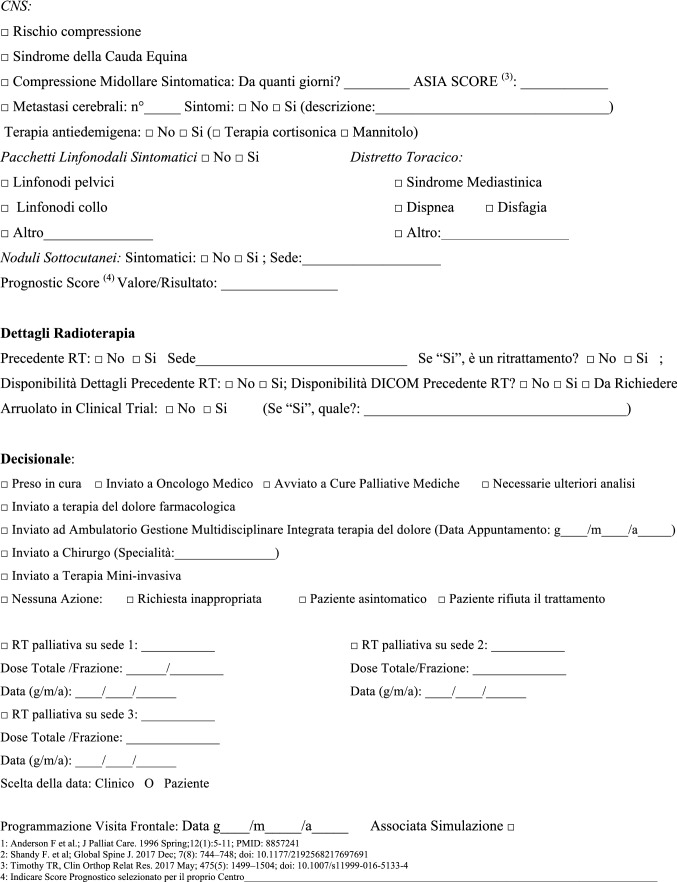
Form for remote-visit for Palliative Radiation Therapy (Italian Version)

The NORMALITY clinical care model aims to make the stays in RT departments of patients dealing with complex logistic settings (e.g., in home care or hospice, living a long distance from the closest RT department) as short as possible. Ideally, patients should receive clinical visits, simulations, and single (or first) PRT delivery on the same day. Single-fraction PRT should be preferred whenever possible, unless the risk of unacceptable toxicity cannot be avoided. This was realized in some fast-track or rapid-response RT programs [12, 51–53]. The integration proposed to such acknowledged care models is to prepare ahead of patient arrival via least two levels of teleconsultation (triage and remote visits). The first level (triage) aims to enable the triage of patients possibly requiring PRT through a simplified information collection method that can be performed by a clinician or a qualified nurse. The triage can subsequently require a remote visit. This second-level contact with the patient (remote visit) involves a single or repeated remote visit, with potentially more in-depth information collected by the RO in order to administer the PRT prescription. If imaging evaluation is needed, the caregiver can be asked to acquire imaging, or alternatively (depending privacy rules), sharing through a computer network could be considered. Teleconsultation for triage and remote visits can be done by interactive and video calls, but also through phone calls that can effectively respond to such needs [12]. Figure 2 represents a form in the English version that facilitates the first triage (the Italian version is shown in Fig. 3). Figure 4 represents a form (in the English version) to facilitate remote visits (the Italian version is shown in Fig. 5). The summary of the PRT indications (i.e.: PRT Normality model summary) for this peculiar setting (not belonging to regular PRT) with the relative consensus is reported in Table 3 for palliative emergencies and Table 4 for palliative non-emergencies. With respect to the *PRT COVID-19 summary*, in PRT Normality model  summary 20 CPIs were identified and one additional subtype clinical presentation was included: non-painful bone metastases. Thirty subtype indications for the 20 CPIs were summarized. The average agreement (“agree” + “strongly agree”) was over the strong agreement threshold (i.e.: 80%), ranging from 82 to 100% among the 30 topic items, of which the inner rate of agreement of the first vote ranged from 33 to 92%.

**Table 3:** PRT Normality Model Summary—Normality model PRT indications: palliative emergencies

Emergencies						
Reference		Main Prescriptive Indication	Alternative	Additional Statement (if any)	% Consensus Vote*	
					A = Agreement (1 + 2)	
					D = Disagreement (3 + 4)	
					SA = Strong Agreement (1)	
					SD = Strong Disagreement (4)	
***E1 Metastatic Epidural Spinal Cord Compression (MESCC)***						
QE1e	AIRO Pall	8 Gy/1fx8Gy (Maranzano [19])	**Preferable for alternative:**	BID option can be considered balancing pt and department’s logistic, being suitable for hospitalized pt but not limited to those only	A = 100% [SA = 92%]	D = 0% [SD = 0%]
			• 20 Gy/5fx4Gy			
			**Secondary alternative option:**			
			• 20 Gy/4fx5GyBID [SHARON project [22, 23]]			
			• 6 Gy/1fx6Gy			
***E2 Hemostasis (including Hemoptysis)***						
QE2h	AIRO Pall	**H&N cancer bleeding:**	**Preferable for alternative:**	**–**	A = 100% [SA = 42%]	D = 0% [SD = 0%]
		• 20 Gy/5fx4Gy	• 20 Gy/4fx5Gy BID			
			[SHARON project [28]]			
			**Secondary alternative option:**			
			• 44.4 Gy/12fx3,7 Gy			
			• 8 Gy/1fx8Gy			
QE2i	AIRO Pall	**Esophageal cancer bleeding:**	20 Gy/5fx4Gy	**–**	A = 100% [SA = 42%]	D = 0% [SD = 0%]
		• 6 Gy/1fx6Gy				
		• 8 Gy/1fx8Gy				
		• 12 Gy/4fx3Gy BID [SHARON project [23]]				
QE2j	AIRO Pall	**Gastric cancer bleeding:**	20 Gy/5fx4Gy	**–**	A = 84% [SA = 42%]	D = 16% [SD = 0%]
		• 6 Gy/1fx6Gy				
		• 8 Gy/1fx8Gy (with anti-emetic)				
QE2l	AIRO Pall	**Pelvic malignancies bleeding:** 8 Gy/1fx8Gy	**Preferable for alternative:**	BID option can be considered balancing pt and department’s logistic, being suitable for hospitalized pt but not limited to those only	A = 100% [SA = 50%]	D = 0% [SD = 0%]
			• 24 Gy/3fx8Gy Day 0, 7, 21 [29]			
			• 18 Gy/4fx4.5 Gy BID [SHARON project [30]]			
			**Secondary alternative option:**			
			• 18 Gy/3fx6Gy (Day 0, 7, 21)			
			• 20 Gy/5fx4Gy			
			• 24 Gy/6fx4Gy			
QE2m	AIRO Pall	**Hemoptysis:**	• 20 Gy/5fx4Gy	**–**	A = 92% [SA = 75%]	D = 8% [SD = 0%]
		• 17 Gy/2fx8.5 Gy (weekly)				
***E3 Mediastinal Syndrome***						
QE3e	AIRO Pall	**Superior vena cava syndrome:**	**Preferable for alternative:**	BID option can be considered balancing pt and department’s logistic, being suitable for hospitalized pt but not limited to those only	A = 100% [SA = 75%]	D = 0% [SD = 0%]
		• 17 Gy/2fx8.5 Gy weekly [MRC] [32, 33]	• 8 Gy/1fx8Gy			
		• 20 Gy/5fx4Gy	**Secondary alternative option:**			
			• 20 Gy/4fx5Gy BID [SHARON project [34]]			

*Consensus Vote: 1 = Strongly Agree; 2 = Agree; 3 = Disagree; 4 = Strongly Disagree

*MESCC* Metastatic Epidural Spinal Cord Compression; *fx* fraction; OS: overall Survival; *RT* Radiotherapy; *pt* patient; *BID* bis in die; *Q* schedule repetition interval; *QoL* quality of life; *SBRT* stereotactic body RT; *mets* metastases; *wks* weeks; *PEG* percutaneous endoscopic gastrostomy; *WBRT* whole brain RT; *TMZ* Temozolamide; *mth* months; *IMRT-SIB* Intensity modulated RT—Simultaneous integrated boost

**Table 4:** PRT Normality Model Summary—Normality model PRT indications: palliative non-emergencies

Palliative (Non-emergencies)						
Reference		Main Prescriptive Indication	Alternative	Additional Statement (if any)	% Consensus Vote*	
					A = Agreement (1 + 2)	
					D = Disagreement (3 + 4)	
					SA = Strong Agreement (1)	
					SD = Strong Disagreement (4)	
**P1 Painful bone metastasis**						
QP1f	AIRO Pall	8 Gy/1fx8Gy	**Preferable for alternative:**	• SHARON project as useful option for painful complicated lesions (i.e.: extraosseous disease, impending fracture, pathological fracture); see also “Section E1”	A = 100% [SA = 75%]	D = 0% [SD = 0%]
			• 20 Gy/4fx5GyBID [SHARON project [22]]	• BID option can be considered balancing pt and department’s logistic, being suitable for hospitalized pt but not limited to those only		
			**Secondary alternative Option:**	• For extreme clinical settings of extensive bone involvement, or retreatment/ pain refractory to pain killers: caution consider “Half-body RT” (i.e.: lumbar + bony pelvis + femurs—15 Gy/4fx3.75 Gy BID [SHARON project [35]]		
			• 20 Gy/5fx4Gy			
***P2 Non-painful bone metastasis***						
QP2b	AIRO Pall	–	–	• Consider to delay RT or evaluate SBRT (depending if oligometastatic and on the basis of prognostic score and impending fracture risk)	A = 100% [SA = 75%]	D = 0% [SD = 0%]
				• Consider RT if impending fracture: if “Yes”, see E1 + P1 + P3		
***P3 Bone Oligometastases Suitable for SBRT***						
QP3d	AIRO Pall	SBRT 1–5 fx (BED 50-60 Gy if not compromising spinal cord constraints)	Single fraction (16 to 24 Gy) SBRT for Retreatment of Symptomatic MESCC	• Apply validated prognostic score before clinical indication	A = 100% [SA = 58%]	D = 0% [SD = 0%]
				• Consider SBRT in case of future risk of MESCC or fracture		
				• Alternatively, consider delay or avoid SBRT, and/or non-SBRT RT indications		
***P4 Retreatment of painful bone metastasis***						
QP4b	AIRO Pall	8 Gy/1fx8Gy	SBRT Single fraction (16 to 24 Gy) SBRT for Retreatment of Symptomatic MESCC	• Waiting a minimum of 6 weeks after completion of the initial RT	A = 92% [SA = 42%]	D = 8% [SD = 0%]
				• For highly selected clinical settings of extensive bone involvement, or retreatment/ pain refractory to pain killers: cautiously consider “Half-body RT” (i.e.: lumbar + bony pelvis + femurs—15 Gy/4fx3.75 Gy BID) [SHARON project [35]]		
***P5 Adjuvant (post-surgery) bone metastasis radiotherapy***						
QP5c	AIRO Pall	30 Gy/10 fx3Gy	• 20 Gy/4fx 5 Gy	• Apply a validated prognostic score for RT to evaluate if expected survival < 3/3–6/ > 6 mth	A = 100% [SA = 75%]	D = 0% [SD = 0%]
			• 20 Gy/5fx 4 Gy	• RT may be postponed in case of asymptomatic pt		
				• RT may be performed secondarily in case of progressive post-operative signs		
				• If Adjuvant RT have been indicated after surgery for MESCC: it should not be postponed over 3–4 weeks		
***P6 Pain NOT associated to Bone Mets**** (e.g.: direct infiltration, primary pancreatic; H&N; Lymph-node infiltrating surrounding structures, etc.)*						
QP6f	AIRO Pall	**H&N**:	**H&N Preferable for alternative:**	BID options (Sharon, QUAD Shot) can be considered balancing pt and department’s logistic, being suitable for hospitalized pt but not limited to those only	A = 100% [SA = 83%]	D = 0% [SD = 0%]
		• 20 Gy/5fx4Gy	• 20 Gy/4fx5GyBID [SHARON project [28]]			
			• 14 Gy/4fx 3.5 Gy BID (repeated Q4 weeks interval × 2 times) [QUAD SHOT- RTOG 8502 [26, 27]			
			**Secondary alternative option:**			
			• 8 Gy/1fx8Gy			
			• 24/3fx8Gy Q 0–7-21 (weekly) [25, 29, 36]			
			• 18/3fx6Gy Q 0–7-21 (weekly)			
QP6g	AIRO Pall	Pain by primary **Gyn + Melanoma + Esophageal + HCC + Pancreas + SCLC/NSCLC:**	o **Gyn + Melanoma**:	–	A = 92% [SA = 33%]	D = 8% [SD = 0%]
		• 8 Gy/1fx8Gy	• 24/3fx8Gy Q 0–7-21			
			• 18/3fx6Gy			
			Q 0–7-21			
			o **Gyn** 18 Gy/4fx4.5GyBID [SHARON project [30]]			
			o **SCLC/NSCLC** [IAEA [37]]:			
			• 16 Gy/2fx 8 Gy (1 week apart)			
			o **Pancreas**			
			10 Gy/1fx10Gy			
***P7 Other than Pain symptoms NOT associated to Bone Mets**** (e.g.: obstruction, etc.)*						
QP7k	AIRO Pall	**H&N**:	**H&N Preferable for alternative:**	• BID options (Sharon, QUAD Shot) can be considered balancing pt and department’s logistic, being suitable for hospitalized pt but not limited to those only	A = 100% [SA = 67%]	*D* = *0% [SD* = *0%]*
		• 20 Gy/5fx4Gy	• 20 Gy/4fx5GyBID [SHARON project [28]]			
			• 14 Gy/4fx 3.5 Gy BID (repeated Q4 weeks interval × 2 times) [QUAD SHOT- RTOG 8502 [26, 27]]			
			**Secondary alternative option:**			
			• 8 Gy/1fx8Gy			
			• 24/3fx8Gy Q 0–7-21 (weekly) [25, 29, 36]			
			• 18/3fx6Gy Q 0–7-21 (weekly)			
			• 30 Gy/6fx6Gy (2 fx/week) [HYPO trial [38]]			
QP7l	AIRO Pall	**Gyn + Melanoma**:	• **Gyn + Melanoma**: 24 Gy/3fx8Gy Q 0–7-21	–	A = 92% [SA = 42%]	D = 8% [SD = 0%]
		• 8 Gy/1fx8Gy	• **Gyn** 18 Gy/4fx4.5GyBID [SHARON project [30]]			
QP7m	AIRO Pall	**Esophageal dysphagia**:	• 12 Gy/4fx3GyBID [SHARON project [23]]	• BID option can be considered balancing pt and department’s logistic, being suitable for hospitalized pt but not limited to those only	A = 100% [SA = 67%]	D = 0% [SD = 0%]
		• 20 Gy/5fx4Gy		• Consider either esophageal stent or percutaneous endoscopic gastrostomy (PEG) tube placement		
QP7n	AIRO Pall	**Pancreas Symptomatic** (non-pain): 10 Gy/1fx10Gy	**Pancreas Symptomatic** (non-pain): 8 Gy/1fx8Gy	–	A = 83% [SA = 33%]	D = 17% [SD = 0%]
QP7o	AIRO Pall	**SCLC:** 16 Gy/2 fx 8 Gy (1 week apart) [IAEA [37]]	**SCLC Preferable for alternative:**	–	A = 100% [SA = 58%]	D = 0% [SD = 0%]
			• 8 Gy/1fx 8 Gy			
			• 20 Gy/4fx5Gy BID [SHARON project [34]]			
			**Secondary alternative option:**			
			• 10 Gy/1fx 10 Gy [IAEA [37]]			
QP7p	AIRO Pall	**NSCLC**:	**NSCLC Preferable for alternative:**	• Order reported for main indication (17 Gy) follows the highest consensus reported in ESTRO-ASTRO Consensus (Guckemberger et al. [5])	A = 100% [SA = 58%]	D = 0% [SD = 0%]
		1.17 Gy/2fx 8.5 Gy (1 week apart) [32, 33]	• 20 Gy/5fx 4 Gy			
		2.8 Gy/1fx 8 Gy	• 20 Gy/4fx5Gy BID [SHARON project [34]]			
			**Secondary alternative option:**			
			• 10 Gy/1fx 10 Gy [IAEA [37]]			
***P8 Symptomatic Haematological Malignancies (non-emergencies)***						
QP8c	AIRO Pall	**Accordingly to** Yahalom [4]:	**Accordingly to Yahalom **[4]	**Accordingly to Yahalom **[4]	A = 100% [SA = 92%]	D = 0% [SD = 0%]
		• **Symptomatic aggressive NHL** (no chemo options) Life expectancy > 3 months 25 Gy/5fx5Gy				
		• **Symptomatic aggressive NHL** (no chemo options) Life expectancy < 3 months: 8 Gy/1fx8Gy				
		• **Symptomatic multiple myeloma** (No cord compression): 8 Gy/1fx8Gy				
		• **Symptomatic multiple myeloma** (Cord compression): 20 Gy/5fx4Gy				
		• **Symptomatic indolent lymphoma** (No cord compression): 4 Gy/1fx4Gy				
		• **Symptomatic indolent lymphoma** (Cord compression): 20 Gy/5fx4Gy				
		• **Myeloid sarcoma/leukemia** -Cranial leptomeningeal disease: 8 Gy/2fx4Gy				
		• **Myeloid sarcoma/leukemia**—Focal leptomeningeal spine disease, and symptomatic chloroma outside the CNS: 12 Gy/3fx4Gy				
***P9 Other Oligometastases Suitable for SBRT (Lung)***						
QP9b	AIRO Pall	**Oligometastases (1–3):**	–	• Consider for pt at prognosis > 6mth (by validated prognostic score)	A = 100% [SA = 58%]	D = 0% [SD = 0%]
		• Lesion ≤ 3 cm surrounded by lung parenchyma: 54 Gy/3fx18Gy		• Consider for pt with disease-free interval ≥ 6 mth		
		• Lesion near chest wall or size > 3 cm: 55 Gy/5fx11Gy		• Biologic effective dose > 100 Gy (if not compromising OAR constraints—AAPM)		
		• Lesion within 2 cm of mediastinum or brachial plexus: 60 Gy/8fx7.5 Gy [SabrComet [39]; SabrComet3 [40]]				
***P10 Other Oligometastases Suitable for SBRT (Liver)***						
QP10c	AIRO Pall	BED ≥ 100 (if not compromising liver parenchyma and other constraints according to AAPM)	o **For small, non-central lesions:**	• Consider for pt at prognosis > 6mth (by validated prognostic score)	A = 100% [SA = 75%]	D = 0% [SD = 0%]
			• 50 Gy/5fx10Gy	• Consider for pt with disease-free interval ≥ 6 mth		
			• 54 Gy/3fx18Gy (every second day) [SabrComet3 [40]]			
			o **For lesions near the biliary tree:** 54 Gy/6fx9Gy			
***P11 Other Oligometastases Suitable for SBRT (Adrenal)***						
QP11b	AIRO Pall	• 40 Gy/5fx8Gy [SabrComet3 [40]]	36 Gy/3fx12Gy	• SBRT 1–5 fx	A = 100% [SA = 50%]	D = 0% [SD = 0%]
		• 35 Gy/5fx7Gy		• Consider for pt at prognosis > 6mth (by validated prognostic score)		
				• Consider for pt with disease-free interval ≥ 6 mth		
				• Evaluate constraints as per AAPM		
***P12 Other Oligometastases Suitable for SBRT (Lymph-node asymptomatic)***						
QP12b	AIRO Pall	• 40 Gy/5fx8Gy [SabrComet3 [40]]	36 Gy/3fx12Gy	• SBRT 1–5 fx	A = 100% [SA = 50%]	D = 0% [SD = 0%]
		• 35 Gy/5fx7Gy		• Consider for pt at prognosis > 6mth (by validated prognostic score)		
				• Consider for pt with disease-free interval ≥ 6 mth		
				• Evaluate constraints as per AAPM [41]		
***P13 Brain metastases (N° 1–4)***						
QP13d	AIRO Pall	**1–4 Brain Mets, good KPS, no extracranial disease**	–	• SRS	A = 100% [SA = 58%]	D = 0% [SD = 0%]
		• 15 Gy/1fx15Gy				
		lesion > 3 cm ≤ 4 cm [42]				
		• 18 Gy/1fx18Gy				
		lesion > 2 cm ≤ 3 cm [42]				
		• 21 Gy/1fx21Gy lesion ≤ 2 cm				
		• 24 Gy/1fx24Gy lesion ≤ 2 cm [42] [RTOG 9508]				
***P14 Brain metastases (N° 5–10)***						
QP14c	AIRO Pall	**5–10 Brain Mets good KPS, no extracranial disease:**	**5–10 Brain Mets, good KPS, no extracranial disease:** Preferable for Alternative: **Palliation WBRT IMRT-SIB** (SIB40 + 30)Gy/10fx(SIB4 + 3)Gy Secondary Option: **Palliation WBRT**	• SRS (if single fraction adopted)	A = 82% [SA = 58%]	D = 8% [SD = 0%]
		• 18 Gy/1fx18Gy	• 30 Gy/10fx3Gy			
		• 15–20 Gy/1fx15-20 Gy				
***P15 Brain metastases**** (N° > 4), poor KPS, meningeal involvement*						
QP15e	AIRO Pall	**Brain metastasis Palliation, poor Prognosis leptomeningeal disease:** 20 Gy/5fx4Gy	**Life expectancy > 3 mth:** 30 Gy/10fx3Gy	**–**	A = 92% [SA = 75%]	D = 8% [SD = 0%]
***P16 Primary symptomatic Brain tumor, poor KPS***						
QP16d	AIRO Pall	**Glioblastoma**	• 34 Gy/10fx3.4 Gy [Malmstrom [45]]		A = 100% [SA = 58%]	D = 0% [SD = 0%]
		• 25 Gy/5fx5Gy [Roa 2015 [46]]				
***P17 Postoperative Brain Mets***						
QP17b	AIRO Pall	**Postoperative SRS of resection cavity:**	• 15-18 Gy/1fx15-18 Gy [Kepka [49]]	**The dose depends on target diameter (in cm):	A = 92% [SA = 50%]	D = 8% [SD = 0%]
		• 20-24 Gy/1fx20-24 Gy [Brown [47]]**	• 25 Gy/5fx5Gy cavities larger than 5 cm [Kepka [49]]	• < 2.0 cm		
		• 16 Gy/1fx16Gy		• ≥ 2 ≤ 2.9 cm ##The dose depends on target size (in cc):		
		• 14 Gy/1fx14Gy		• ≤ 10 cc		
		• 12 Gy/1fx12Gy [Mahajan [48]]##		• 10.1–15 cc		
		**Postoperative fractionated SRT of resection cavity:**		• > 15 cc		
		• 35-25 Gy/5fx7-5 Gy				

*Consensus Vote: 1 = Strongly Agree; 2 = Agree; 3 = Disagree; 4 = Strongly Disagree

*MESCC* Metastatic Epidural Spinal Cord Compression; *fx* fraction; OS: overall Survival; *RT* Radiotherapy; *pt* patient; *BID* bis in die; *Q* schedule repetition interval; *QoL* quality of life; *SBRT* stereotactic body RT; *mets* metastases; *wks* weeks; *PEG* percutaneous endoscopic gastrostomy; *WBRT* whole brain RT; *TMZ* Temozolamide; *mth* months; *IMRT-SIB* Intensity modulated RT—Simultaneous integrated boost

The latest versions of such materials can also be retrieved in the following section of the “La Rete del Sollievo” (NOS) website (http://www.gemelliart.it/laretedelsollievo/retedelsollievo-modelliassistenziali/). An interactive list of Italian RT departments providing different palliative services endorsed by the AIRO can also be downloaded from this website.

## Discussion

Our paper aims to deal with three main issues regarding PRT: how to choose a PRT prescription during the COVID-19 pandemic; highlight the priority of administering PRT for patients both during the COVID-19 pandemic and in the future; and how to manage the risk of underuse of PRT in the future in patients dealing with complex logistic scenarios (particularly after the emergency pandemic experience, which suggests that different approaches to PRT are preferable if the RT department is inaccessible).

How to choose a PRT prescription during the COVID-19 pandemic? The COVID-19 pandemic challenges healthcare systems worldwide [1, 54]. Some authors described that two phases are possibly expected from an RO’s perspective: an early phase in which the department’s human resources are not limited and potentially all treatments could be provided for patients; and a second phase (“late phase or scenario”) in which the infection spread could limit the global amount of actually deliverable RT treatments [5]. To address this scenario, hypofractionated RT schedules have been proposed to reduce the number of contacts while effectively treating patients [4–7]. A recent survey reported by Jereczek-Fossa et al*.* confirmed that in a highly impacted country like Italy, 73.6% of RT departments shifted to adapted hypofractionated RT schedules [55]. To the best of our knowledge, most guidelines have not focused on PRT, apart from one addressing bone metastases [8]; thus, an RO needing to prescribe PRT during the COVID-19 pandemic would find indications distributed across different papers. The proposed PRT COVID-19 summary (Tables 1 and 2) could aid ROs during this period. The limitation that there are papers missing in our literature search because they were published after our search was conducted exists; however, the average support the summary provides for readers is not compromised and the expert consensus vote poses additional utility, offering an overview perspective for interpreting other similar indications.

What is the priority of administering PRT for our patients, both during the COVID-19 pandemic and in the future? Prioritization of PRT has been an object of discussion during the COVID-19 pandemic period [3, 9]. Yerramilli et al*.* suggested prioritizing PRT only for emergencies, providing a triage model to check for the need for PRT [9], although some authors have raised concerns over this [10]. Conversely, Tagliaferri et al*.* suggested a more inclusive triage-based patient selection strategy, possibly providing PRT even to COVID-19 positive patients, despite the consideration of dealing with highly aggressive diseases such as melanoma [56]. Neither considered the different phases of infection spread within the RT department. Van de Haar et al*.* [3] suggested multiple phases detailing the steps of expectable crisis from the clinical perspective of RT, surgical, and medical oncology departments, but still did not indicate if or how passing through one of the mentioned level of crisis to another would change the priority list that puts PRT at level four of five in their paper. A few authors [5, 6] indicated that different RT and PRT schedules are deliverable in the early or later (more complex) phases (both are included in Tables 1 and 2).

We substantially agreed with the average concern of treating patients during the COVID-19 period. Moreover, most of the indications provided in the early pandemic were unaware of the possible consequent scenarios, thus preparing ahead for the worst possible scenario. A wide range of consequences have been described for RT departments, ranging from compromised [57] to more manageable [58]. In our opinion (when indicated), PRT should remain one of the highest priority treatments from the perspective of ROs. For the two major oncological aims (cure and palliation), the pursued outcomes, as measured by the most appropriate endpoint (i.e., overall survival (OS) for cure and quality of life (QoL) for palliation, respectively), are equivalent from the patient’s perspective. Until we are not forced to restrict the delivery of RT to our patients due to the risk of infective spread, palliative and curative settings should be equally prioritized [11]. To put this into context, consider an example on bone-related pain control: if RT is not administered when indicated, the possibly needed dose escalation of medical analgesic therapy can determine side effects affecting QoL, despite controlling pain levels (besides the cost-effective impact on health services by increased drug administration). Separate administration of either palliative RT or medical analgesic therapy should not be considered equivalent by ROs; the concomitant integration of both with modulation over time should be the gold standard.

How to manage the risk of underuse of PRT in the future in patients dealing with complex logistic scenarios? In the future, when the COVID-19 pandemic is over, the issue of patients suitable for PRT who are dealing with complex logistic settings and who are at risk of losing their chance of receiving relief by PRT will surely persist. Looking at the current experience of emergency departments, we are afraid that PRT could be replaced by medical or different alternatives if it is not easily and logistically manageable. Some of the indications provided for PRT during COVID-19 pandemic can be safely and effectively maintained in these peculiar settings. The NORMALITY clinical care model aims to enhance the chance that these patients receive acceptable compromises, aiming for an efficient PRT schedule. The combination of clinical visits, simulation, and RT delivery on the same day is a well-known practice that has diffused over several RT centers for at least 30 years. The Rapid Response Radiotherapy Program (RRRP) was proposed in the literature in the 1990s by the Canadian group Chow et al*.* [51–53]. Similarly, the Vancouver Rapid Access (VARA) for incurable lung cancer was presented by Lefresne et al*.* [59], as well as the rapid multidisciplinary management of bone metastases described by Donato et al*.* [60]. The positive impact of the described “advanced practice radiation therapists” on workflows was also explored, for instance, by Job et al*.* [61, 62]. Our model integrates such experiences while focusing on patients with complex logistics, proposing the set of normality model PRT indications for such peculiar settings, as summarized in Tables 3 and 4. If appropriately selected for patients, treatment alternatives such as single fraction treatments applied in emergencies as suggested by Maranzano et al*.* [20], the use of single repeated schedules as per the “0–7–21” PRT schedule proposed by Nguyen et al*.* [26], or the *bis-in-die* (BID) schedules advised by both the “Quad Shot RTOG 8502–QUAD SHOT” report created by Spanos et al*.* [27, 28] and the “Sharon project” for multiple palliative settings [31], can be highly useful. Moreover, our NORMALITY model suggests and offers forms to facilitate the enhancement of preliminary teleconsultations before the first clinical visits of the patients (Figs. 2 and 4). This is in line with the literature acknowledging the efficacy of phone calls [12] and the renewed indication for teleconsultation during the COVID-19 period [9, 14]. Some issues remain unaddressed, including the management of patients strictly requiring hospital admittance and the role of technology in balancing urgent palliative patients’ needs. Clearly, improving such settings will require multidisciplinary collaboration among operators with different specializations and backgrounds dealing with palliation and oriented to facilitate each other’s respective roles and peculiarities.

## Conclusion

We provide a comprehensive summary of the literature guideline indications for PRT during the COVID-19 pandemic along with the respective reference and consensus evaluation voted by the AIRO panel. We also propose a clinical care model (based on the clinical guideline indications provided during the COVID-19 pandemic) including clinical indications and written forms facilitating two levels of teleconsultation (triage and remote visits) in order to evaluate patients for indications for PRT ahead of planning live clinical visits. The normality model could facilitate the provision of PRT to patients dealing with future complex logistic scenarios.

## Appendix

### Medline search strategy

(("radiotherapy"[Subheading] OR "radiotherapy"[All Fields] OR "radiotherapy"[MeSH Terms]) OR ("radiotherapy"[Subheading] OR "radiotherapy"[All Fields] OR ("radiation"[All Fields] AND "therapy"[All Fields]) OR "radiation therapy"[All Fields] OR "radiotherapy"[MeSH Terms] OR ("radiation"[All Fields] AND "therapy"[All Fields]) OR "radiation therapy"[All Fields]) OR ("radiation oncology"[MeSH Terms] OR ("radiation"[All Fields] AND "oncology"[All Fields]) OR "radiation oncology"[All Fields]) OR Palliative[All Fields] OR (Palliative[All Fields] AND ("radiotherapy"[Subheading] OR "radiotherapy"[All Fields] OR "radiotherapy"[MeSH Terms]))) AND (("COVID-19"[All Fields] OR "COVID-2019"[All Fields] OR "severe acute respiratory syndrome coronavirus 2"[Supplementary Concept] OR "severe acute respiratory syndrome coronavirus 2"[All Fields] OR "2019-nCoV"[All Fields] OR "SARS-CoV-2"[All Fields] OR "2019nCoV"[All Fields] OR (("Wuhan"[All Fields] AND ("coronavirus"[MeSH Terms] OR "coronavirus"[All Fields])) AND (2019/12[PDAT] OR 2020[PDAT]))) OR SARS-COV2[All Fields]).
